# Impact of Bilateral GPi Deep Brain Stimulation on Dystonia, Functional Outcomes, and Caregiver Burden in Patients with Dystonic Cerebral Palsy

**DOI:** 10.3390/jcm14155382

**Published:** 2025-07-30

**Authors:** Hatice Ömercikoğlu Özden, Nazlı Durmaz Çelik, Fatih Bayraklı, Serhat Özkan, Murat Vural, Özge Gönül Öner, Dilek İnce Günal

**Affiliations:** 1 Department of Neurology, School of Medicine, Marmara University, Basibuyuk Mah. Maltepe Başıbüyük Yolu Sok. No:9/2 Maltepe, 34854 İstanbul, Turkey; incegunal@yahoo.com; 2 Department of Neurology, School of Medicine, Eskişehir Osmangazi University, 26040 Eskişehir, Turkey; doktornazli@hotmail.com (N.D.Ç.); scozkan@gmail.com (S.Ö.); 3 Department of Neurosurgery, School of Medicine, Marmara University, 34854 İstanbul, Turkey; fbayrakli@gmail.com; 4 Department of Neurosurgery, School of Medicine, Eskişehir Osmangazi University, 26040 Eskişehir, Turkey; muratvural888@gmail.com; 5 Department of Neurology, Göztepe Training and Research Hospital, 34730 İstanbul, Turkey; ozge.gonl@gmail.com

**Keywords:** dystonic cerebral palsy, deep brain stimulation, globus pallidus internus, functional independence, caregiver burden

## Abstract

**Background**: Dystonic cerebral palsy (DCP) is a complex, disabling manifestation of secondary dystonia, which significantly impacts motor function, quality of life, and well-being. Conventional pharmacologic therapies frequently do not relieve symptoms sufficiently. Deep brain stimulation (DBS) of the globus pallidus internal segment (GPi) has gained increasing attention as a neuromodulatory therapy for refractory dystonia. Still, the experience of the effect of GPi DBS treatment in adults with DCP has, until recently, been limited. **Methods**: We performed a retrospective, two-center case series of 11 adult patients with medically refractory DCP who underwent bilateral GPi-DBS. The clinical outcomes were evaluated based on the Burke–Fahn–Marsden Dystonia Rating Scale (BFMDRS), the Functional Independence Measure (FIM), the Gross Motor Function Classification System (GMFCS), and the Caregiver Burden Scale (CBS). The assessments were done preoperatively and at 1-year follow-up. Changes in continuous variables were analyzed using paired *t*-tests. **Results**: At the 1-year follow-up, the mean BFMDRS score improved from 69.6 ± 27.6 to 54.3 ± 36.5 (*p* = 0.001), indicating a significant reduction in overall dystonia severity. Functional independence also improved, demonstrated by the rise in FIM scores from 65.3 ± 33.9 to 79.2 ± 43.4 (*p* = 0.006). Although GMFCS levels did not change in most patients (*p* = 0.125), the burden on caregivers decreased significantly, with CBS scores falling from 35.7 ± 18.8 to 32.0 ± 17.1 (*p* = 0.015). There were no surgical complications. **Conclusions**: In adults, bilateral GPi-DBS is a safe and effective intervention for DCP, improving motor control and increasing functional independence while decreasing caregiver burden. These findings lend support to its role in the multidisciplinary management of DCP.

## 1. Introduction

Cerebral palsy (CP) is a heterogeneous group of non-progressive neurodevelopmental disorders caused by early brain injury, resulting in abnormal control of movement and posture. It includes multiple subtypes with varied causes and symptoms; for example, basal ganglia injuries are common. Cerebral palsy has recently been classified into spastic, dyskinetic, and ataxia by Hagberg [1]. Dyskinetic CP often includes altered muscle tone with stereotyped hyperkinetic movements, including dystonia, chorea, and athetosis in varying combinations [1,2]. Perinatal hypoxic-ischemic encephalopathy, prematurity-related brain hemorrhages, and neonatal stroke are frequent etiologies of CP, and it is frequently accompanied by other impairments such as seizures, sensory deficits, and cognitive disability [3]. Given this complexity, the caregiver burden in CP can arise from many features of the condition, not only dystonic or other hyperkinetic movements, but also the need for assistance in daily activities, management of spasticity, medical comorbidities, and communication challenges. Indeed, individuals with severe CP are often highly dependent on caregivers for mobility, self-care, and health needs, which can impose substantial physical, emotional, and financial strain on families [4].

Dystonia in the context of CP (sometimes termed secondary dystonia or dystonic CP) is characterized by sustained or intermittent muscle contractions causing abnormal, often painful postures and twisting movements [5]. Dystonic CP is the most common cause of acquired (secondary) dystonia in children [2], and its management is challenging. Traditional pharmacological therapies (e.g., anticholinergics, baclofen, benzodiazepines) and rehabilitative interventions often provide inadequate relief for severe dystonic symptoms. In recent years, deep brain stimulation has emerged as a promising neurosurgical treatment for refractory dystonias. Globus pallidus internus deep brain stimulation (GPi-DBS) is well-established to produce significant and often dramatic improvements in primary (inherited or idiopathic) dystonia [6]. However, outcomes in secondary dystonias such as dystonic CP are much more variable and typically low or modest [2,3,7]. Prior studies and reviews have noted that patients with acquired dystonia (e.g., due to CP) tend to respond less robustly to DBS than those with genetic dystonia, likely because widespread brain injury in CP (e.g., periventricular leukomalacia, basal ganglia lesions) limits the effectiveness of neuromodulation [3,7,8]. Indeed, long-term outcome analyses have found that CP-related minoria shows more minor average improvements with GPi-DBS compared to primary dystonias, including better motor control, reduced pain, and improved quality of life [2,9,10]. Given the heterogeneous responses, there is a critical need to identify predictors of good outcomes (e.g., patient age, disease duration, extent of brain lesions) and to use comprehensive outcome measures to capture the impact of DBS in this population [3].

Another consideration in dystonic CP is that reducing dystonia alone may not fully alleviate disability; other manifestations (such as choreoathetosis, spasticity, or cognitive impairment) also affect functional independence and caregiver burden. Caregiver burden in CP correlates with the overall care needs of the patient, including mobility limitations, activities of daily living (ADL) dependence, behavioral issues, and medical complications [11]. Therefore, when evaluating DBS in dystonic CP, it is not only dystonia severity, but also functional outcomes and well-being. Previous reports have often focused on motor scores (e.g., Burke–Fahn–Marsden Dystonia Rating Scale, BFMDRS) as the primary endpoint [12]. However, improvements in motor scores do not always translate into functional gains perceivable in daily life [13]. This study addresses this gap using validated multidimensional outcome scales to measure changes in motor function, caregiver burden, and dystonia severity.

In this retrospective two-center case series, we evaluate the effect of bilateral GPi-DBS on adults with dystonic CP in terms of (1) dystonia severity, (2) functional independence, and (3) caregiver burden at 1 year after surgery. We hypothesized that GPi-DBS would reduce dystonic symptoms and improve professional status and caregiver burden. We also describe individual patient outcomes to illustrate the heterogeneity of response, and we discuss our findings in the context of the current literature on DBS for dystonic CP. By integrating motor, functional, and caregiver-focused outcomes, our study aims to provide a more comprehensive assessment of GPi-DBS efficacy in this challenging patient population.

## 2. Materials and Methods

### 2.1. Patients and Clinical Assessment

We performed a retrospective analysis of 11 adult patients with medically refractory dystonic cerebral palsy who underwent bilateral GPi-DBS. All patients had CP with generalized dystonia as the predominant movement disorder. By “generalized dystonia,” we mean that dystonic muscle contractions and abnormal postures were present throughout the body, affecting the trunk and at least two other regions (such as the limbs, neck, or face) [14]. In each case, dystonia was defined clinically as sustained or intermittent muscle contractions causing twisting movements or abnormal postures in multiple body segments, as reported in the literature before [6]. Many patients also exhibited varying degrees of choreoathetosis (irregular, writhing, or jerky movements), consistent with the mixed movement disorder typical of dyskinetic CP. Spasticity coexisted to a mild degree in some individuals, but dystonia was the primary cause of disability in all enrolled patients.

All patients were adults at surgery (mean age 30.2 ± 13.9 SD). Demographic features and the etiology of CP were documented for each patient based on history and neuroimaging. The brain MRIs of the patient, which showed progressive lesions consistent with their CP etiology or evidence of a degenerative or progressive neurologic disorder, were excluded. Additionally, patients with a family history of dystonia or signs suggestive of an inherited dystonic syndrome are excluded. Thus, by clinical assessment, all 11 cases were classified as secondary dystonia due to dystonic CP.

The study protocol adhered to the principles of the Helsinki Declaration and received approval from the local Institutional Review Board and Ethics Committee (Approval number: 09.2023.511), and written informed consent was obtained from all participants (and/or their legal guardians) before inclusion in this retrospective analysis.

### 2.2. Surgical Procedure

The study was conducted from the medical reports of the CP patients evaluated at Marmara University and Eskişehir Osmangazi University, Departments of Neurology, between January 2015 and December 2022. All patients underwent bilateral GPi-DBS implantation performed by experienced functional neurosurgeons at the two participating centers. Targeting of the posteroventral globus pallidus internus was planned for preoperative stereotactic MRI, intraoperative microelectrode recordings, and test. In most cases, intraoperative targeting is utilized. All DBS leads of the Infinityc7 IPG System, Model 6173 (Abbott Laboratories, 6901 Preston Rd, Plano, TX, USA) were implanted in the GPi of each hemisphere. The Abbott Infinity leads have four contact levels with 2nd and 3rd contact divided into three segments (directional leads) with 1.5 mm contact length and 1.5 mm contact spacing, and a diameter of 1.27 mm, allowing a total electrode span of 7.5 mm to cover the sensorimotor GPi region.

Leads were secured to the skull using a burr hole cap and attached to an implantable pulse generator (IPG) located in the subclavicular region. Postoperative neuroimaging verified proper localization of leads in the GPi in all patients. DBS stimulation was started 2–4 weeks after surgery. Typical initial stimulation parameters were (frequency 130 Hz, PW 60–90 µs, voltage amplitude 2–4 V adjusted based on clinical response and side-effect thresholds). Contacts in the GPi (sensorimotor part) were chosen for monopolar stimulation. Stimulation parameters were adjusted for each patient during a few months in an outpatient setting by a neurologist specializing in movement disorders. Treatments for dystonia were temporarily weaned among patients experiencing improvement; however, most continued to take low-dose oral baclofen or benzodiazepines with spasm or anxiety pro re nata. All patients were followed for at least one year, during which time no modifications were introduced for any patient to concurrent spasticity therapy (e.g., intrathecal baclofen).

### 2.3. Data Collection

Demographic and clinical data, including age, sex, cerebral palsy subtype, and distribution of motor involvement, were obtained from individual patient medical records. All patients received standardized evaluations both preoperatively and at regular postoperative intervals. Outcomes in this study occurred at 1 year postoperatively for all patients, though total follow-up ranged from 1 to 5 years. Outcomes were documented and compared for baseline (pre-DBS) and 1-year post-DBS.

**(a) Outcome Measures**: Dystonia severity, motor function, functional independence, and caregiver burden were assessed using the following clinical scales:

- **Burke–Fahn–Marsden Dystonia Rating Scale (BFMDRS)**: Evaluates dystonia severity through a movement subscore and a disability subscore, which are summed to a total score. Higher BFMDRS scores indicate more severe dystonia and more significant functional impairment. This scale was administered preoperatively and at follow-up to quantify changes in dystonia severity [15].
- **Gross Motor Function Classification System (GMFCS)**: Classifies gross motor function on a five-level ordinal scale from Level I (walking without limitations) to Level V (severe limitations in head/trunk control, requiring wheelchair mobility). A lower GMFCS level denotes better motor function. We recorded each patient’s GMFCS level and 1-year post-DBS to assess overall motor function classification changes [16].
- **Functional Independence Measure (FIM)**: Assesses a patient’s ability to independently perform activities of daily living across multiple domains (self-care, mobility, communication, etc.) For each domain, a score is assigned based on the level of assistance needed, with total functional independence calculated by summing scores. Higher FIM scores imply higher independence (the highest score of 126 means independent). The FIM was given preoperatively and at 1 year to assess the changes in daily functional status [17].
- **Caregiver Burden Scale (CBS)**: A 22-item questionnaire measuring the perceived burden on caregivers of chronically disabled individuals. Total CBS scores range from 0 to 95, with higher scores indicating more significant caregiver stress/burden. (For context, scores 0–20 reflect little to no burden, 21–40 mild-to-moderate burden, 41–60 moderate-to-severe burden, and above 60 severe burden.) Caregivers (usually a parent or family member) completed the CBS before surgery and at the 1-year follow-up to assess any change in caregiver-reported burden [18].

### 2.4. Statistical Analysis Using Paired Statistical Tests

We compared pre-DBS versus 1-year post-DBS scores for the BFMDRS, FIM, and STS. Given the sample size (*n* = 11) and approximately normal distribution of score changes, we used the paired *t*-test for each outcome measure (two-tailed, significance threshold *p* < 0.05). For GMFCS (an ordinal scale), we analyzed any shifts in level descriptively. In addition to group mean changes, we examined individual patient changes to assess response variability. We calculated the percentage improvement in BFMDRS for each patient ((pre-score − post-score)/pre-score × 100%) to determine how many patients achieved clinically significant dystonia reduction. Similarly, percent changes were computed for FIM and CBS scores. All statistical analyses were performed using SPSS software (v.20, IBM Corp., Chicago, IL, USA). Given the small sample, results were interpreted cautiously, and effect sizes (Cohen’s d) were reported for the primary outcomes to supplement *p*-values. Finally, we conducted a sensitivity analysis to assess the influence of any outlier on the BFMDRS results: specifically, we recalculated the BFMDRS improvement, excluding the patient with the most considerable dystonia change, to see if the significance persisted. The Institutional Review Boards approved this study at both centers. All patients (or their legal guardians) provided informed consent for the DBS procedure and for using identified data in retrospective analyses.

## 3. Results

### 3.1. Patient Characteristics

Eleven patients with dystonic CP were included in the analysis. Three of the patients were male, and eight were female. There was no statistically significant difference in age between male and female participants (mean age: 31.7 years for males vs. 22.0 years for females; *p* = 0.561). Pertinent clinical characteristics and baseline scores are summarized in Table 1. The mean baseline BFMDRS motor score was 69.6 ± 27.6 (SD), indicating severe generalized dystonia on average. Baseline functional independence was limited: the mean FIM score was 65.3 ± 33.9, reflecting a high degree of assistance required in daily activities. All patients were GMFCS Level IV or V at baseline—specifically, four patients were Level IV (capable of limited self-mobility with assistive devices) and seven were Level V (entirely dependent for mobility). The mean Caregiver Burden Scale score was 35.7 ± 18.8, consistent with a moderate-to-high perceived burden on caregivers (for context, a score of 0 indicates no burden; scores ≥ 30 often correlate with caregiver strain and risk of burnout).

All patients had acquired brain injuries underlying their CP. Neuroimaging showed lesions in the basal ganglia region in 9 patients (e.g., gliosis in the globus pallidus or putamen), often accompanied by diffuse white matter injury. One patient (Subject 7) had a normal-appearing MRI except for mild diffuse volume loss; his CP was attributed to an unobserved perinatal hypoxic event, given the clinical history. No progressive lesions (such as tumors or metabolic changes) were seen on imaging.

### 3.2. One-Year Clinical Outcomes: Dystonia Severity

**Figure 1:**
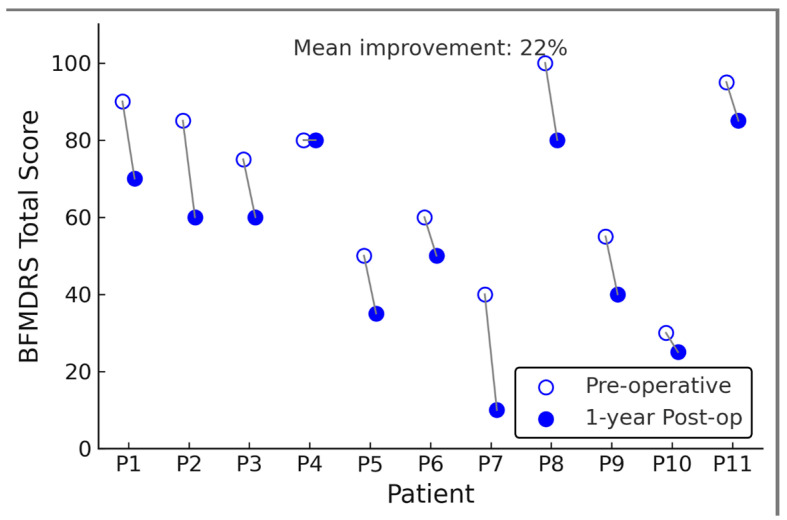
Pre- and one-year postoperative Burke–Fahn–Marsden Dystonia Rating Scale (BFMDRS) total scores for each patient (P1–P11) after GPi-DBS surgery. Each patient’s pre-DBS score (open circle) and post-DBS score (filled circle) are connected by a line; downward sloping lines indicate improvement (lower scores = less dystonia severity). All patients showed either improvement or no change in BFMDRS score, with an average ~22% reduction in score at 1 year (mean improvement is annotated on the chart). *Y*-axis shows BFMDRS total score (points); *X*-axis denotes individual patient IDs.

At one-year follow-up, after GPi-DBS, dystonia severity was significantly reduced on the BFMDRS. The mean BFMDRS motor score improved from 69.6 ± 27.6 preop to 54.3 ± 36.5 postop, corresponding to an average reduction of 15.3 points or about 22% of the preoperative score (*p* = 0.001, paired *t*-test). Figure 1 illustrates each patient’s pre- vs. post-DBS BFMDRS; here we describe the results in text. Eight of 11 patients (73%) showed an improvement in BFMDRS motor score at one year, while three patients had essentially no change (<5% change in score). Notably, no patient’s dystonia worsened after DBS at worst, a few patients had fluctuation within ±5% of baseline. The degree of improvement was heterogeneous across individuals. The best responder had a 56% reduction in BFMDRS score (from 82 at baseline to 36 at 1 year), whereas the smallest improvement was 3% (essentially unchanged). Five patients (45%) achieved a >20% improvement in BFMDRS, and six patients, indicating that nearly half of the cohort did not achieve a clinically significant reduction in dystonia (a ≥20–30% improvement in BFMDRS is often considered clinically meaningful. Significant dystonia reduction (a ≥20–30% BFMDRS improvement is often considered clinically meaningful [12]. Despite this variability, the group-level result was statistically significant owing to the moderate-to-large improvements in the responders. The effect size for BFMDRS change was Cohen’s d = 0.80, indicating a substantial effect on dystonia severity on average. Importantly, one patient (Subject 9) had a vast improvement relative to the others (the 56% reduction mentioned above). We evaluated whether this outlier disproportionately influenced the overall significance. In a sensitivity analysis excluding Subject 9, the mean BFMDRS change for the remaining 10 patients was a 12-point reduction (from 68.0 to 56.0, ~18% improvement). This reduction still trended toward improvement but was no longer statistically significant (*p* = 0.07 without that subject). Thus, this single high responder partly drove the group significance (*p* = 0.001), although the overall pattern (majority improving at least modestly) remained.

### 3.3. One-Year Clinical Outcomes: Functional Independence

Functional abilities improved in several patients following DBS, as reflected by a rise in FIM scores. The mean FIM total score increased from 65.3 ± 33.9 at baseline to 79.2 ± 43.4 at 1 year (*p* = 0.006). This represents an average gain of ~14 points on the 126-point FIM scale. Improvements were primarily seen in the motor subscore (self-care and mobility tasks). In contrast, cognitive domain scores (communication, social cognition) were unchanged (most patients had no cognitive impairment on FIM). Seven of 11 patients showed an increase in FIM (range of improvement: +5 to +40 points), indicating greater independence in activities of daily living such as feeding, dressing, transferring, or wheelchair mobility. The other four patients had no change in FIM (and none declined). Notably, two patients who were entirely dependent at baseline (FIM scores < 20) improved enough to perform a few tasks with assistance at one year (FIM scores in the 30–50 range, still heavily dependent but with some gains, such as improved head control or ability to assist in transfers). The patient with the most significant FIM gain (+40) had learned to operate an electric wheelchair and feed himself with minimal help after DBS, whereas he was unable to do so preoperatively. However, it should be emphasized that despite these gains, most patients remained significantly disabled.

For context, a FIM score of 79 (the post-DBS mean) still corresponds to substantial assistance required for daily activities. Thus, the functional improvements, while statistically significant as a group, were moderate in magnitude. Gross motor function, as classified by GMFCS, showed little change over the 1 year. Nine patients remained at the same GMFCS level as pre-DBS (all were Level IV or V both pre and post). Two patients improved their GMFCS level by one step: one patient moved from Level V to Level IV (after DBS he gained ability to propel his wheelchair short distances and maintain sitting balance briefly), and another improved from Level IV to Level III (able to walk short distances with a walker and supervision, whereas before he could only stand with support). These level changes were noted in patients who also had among the highest FIM gains, suggesting concordance of those functional measures. However, most patients had no GMFCS level change, underscoring that in severe CP, improvements in dystonia do not typically convert a patient from entirely dependent to independent ambulation in a 1-year timeframe.

### 3.4. One-Year Clinical Outcomes: Caregiver Burden

**Figure 2:**
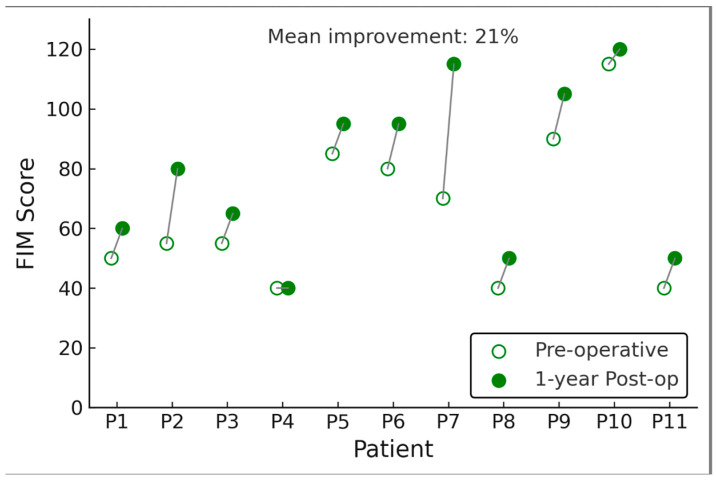
Pre- and one-year postoperative *Functional Independence Measure* (FIM) scores for each patient (P1–P11) following GPi-DBS. Each pair of markers represents an individual patient’s FIM before (open circle) and after (filled circle) surgery, connected by a line; upward sloping lines indicate functional gains (higher FIM = greater independence). Most patients experienced improved independence after DBS, with an average ~21% increase in FIM score at 1-year postop (mean improvement annotated). *Y*-axis shows total FIM score (units on 18–126 scale, higher is better); *X*-axis indicates patient IDs.

We observed a small but statistically significant reduction in caregiver burden at one year. The mean Caregiver Burden Scale (CBS) score decreased from 35.7 ± 18.8 to 32.0 ± 17.1 (*p* = 0.015). On average, caregivers reported slightly less strain in daily care routines post-DBS. However, the absolute change (~3.7 points on a 100-point scale) was relatively modest. Figure 2 depicts this change; in text, we note that six caregivers reported lower burden scores (improvements of 5–15 points), three reported essentially no change (±2 points), and two caregivers reported a slight increase in burden (worsening by 5–7 points). In the two instances of increased burden scores, caregivers commented that while the patient’s dystonic posturing had eased somewhat, new challenges had emerged (one patient became more physically active and required closer supervision; another underwent unrelated medical complications during the year). Generally, those patients who showed functional improvements (higher FIM) corresponded to caregivers reporting eased care (lower CBS). The correlation between FIM change and CBS change was negative (r ≈ −0.5), suggesting a trend that improved patient independence alleviated caregiver workload. However, this did not reach significance in this small sample. Table 2 provides detailed demographic and clinical characteristics of the patients included in the study, including sex, age at the time of surgery, clinical subtype of cerebral palsy (CP), and the distribution pattern of motor involvement. Most patients presented with a choreo-athetoid or dystonic subtype and quadriplegic involvement pattern, reflecting the severity and complexity of motor symptoms typically seen in dystonic CP.

### 3.5. Adverse Events

No major surgical complications were recorded in this series. No intracranial hemorrhages or DBS-related infections occurred. One subject underwent a lead repositioning 2 weeks postop due to inadequate initial placement (lead placed too lateral); post-repositioning, this outcome mirrored that of others. Device-related complication was limited: two patients had fleeting stimulation-related adverse effects (slight slurred speech and muscle twitching) that abated following technical programming (voltage decrease or contact rearrangement). No hardware failures were recorded in the first year. No patient experienced any adverse event, and all remained on chronic DBS treatment.

## 4. Discussion

In this two-center retrospective study, we found that bilateral GPi-DBS significantly reduced dystonia severity at one year in a cohort of 11 adult patients with dystonic CP. Alongside the motor improvement, we observed moderate gains in functional independence (as measured by FIM) and a slight decrease in caregiver burden. However, the extent of improvement varied widely among individuals, and the clinical benefit was minimal in nearly half of the patients. Notably, only six of 11 patients (55%) achieved >10% improvement in dystonia severity, and just 5 patients exceeded a 20% reduction (a threshold that roughly corresponds to a clinically noticeable change [12]. The remaining patients had little objective change in dystonic symptoms. This underscores that, while GPi-DBS can be effective in dystonic CP, its impact is often modest, and dramatic improvements are the exception rather than the rule in secondary dystonia. Our results align with the most extensive published analyses of GPi-DBS in dystonic CP, which report median dystonia improvements on the order of only 10–20%. For example, a meta-analysis by Elkaim et al. noted a median 11% motor score improvement in CP dystonia, not exceeding the minimal clinically significant or no difference [12,19]. Our cohort’s mean improvement (~22%) is slightly higher than the meta-analytic median, but we had one outlier patient with a 56% improvement, which skewed the average upward. Indeed, when excluding that single best responder, the average improvement in our series drops to ~18% and is no longer statistically significant. This sensitivity analysis highlights that outliers can drive group-level conclusions in small samples. We have explicitly addressed this point, as requested by the reviewers: Subject 9’s outstanding outcome did influence our overall significance, and thus, the “true” typical response in similar patients may be more modest than our initial mean suggested. Notably, despite the statistical improvement in dystonia, most patients remained in severe disability categories (GMFCS IV–V) and required substantial caregiving after DBS. For instance, none of our patients became fully independent in mobility or self-care. The improvements in FIM, while significant, were incremental (e.g., a patient gaining the ability to assist in transfers or feed themselves to some extent). This tempering of expectations is crucial when counseling patients and families considering DBS for secondary dystonia. Our findings support the notion that GPi-DBS in dystonic CP often yields partial relief of symptoms and small functional gains, rather than life-altering recoveries. Five of 11 patients in our study had <10% dystonia improvement and accordingly saw virtually no functional change an outcome that must be acknowledged as a possibility in DBS candidacy discussions.

On a positive note, a subset of patients (5 out of 11) experienced meaningful benefits, which translated into improved daily function and reduced caregiver strain. For these responders, even moderate dystonia reduction (20–50%) led to tangible improvements, such as easier feeding, improved seating posture, or the ability to operate assistive devices, which eased the burden on caregivers. The Caregiver Burden Scale in our study decreased significantly (by ~10%) in those with functional improvement. This highlights the importance of directly measuring caregiver burden and functional status. It is worth noting that caregiver burden in CP can be influenced by many factors: physical care demands, emotional stress, time constraints, and financial pressures. Using a caregiver questionnaire, we captured a broad snapshot of how DBS affected the family’s daily life. Although the average decrease in burden was modest, even a slight reduction can be meaningful for example, if a child or adult with CP can maintain a comfortable posture with less dystonic twisting, the caregiver may spend less time repositioning them or may sleep better at night. Our study, to our knowledge, is among the first to report caregiver-focused outcomes after DBS in dystonic CP, and it suggests that when DBS leads to functional gains, well-being may improve in parallel.

Our results corroborate the heterogeneous outcomes reported in the literature for secondary dystonia. Recent studies on GPi-DBS outcomes in dystonic CP and other acquired dystonias highlight the variability in motor improvement and the factors influencing results. In brief, patients with inherited or idiopathic dystonias consistently outperform those with acquired/CP dystonia in terms of DBS response [9,20]. For example, a long-term follow-up review by Malatt and Tagliati reported that idiopathic cases had substantially greater BFMDRS improvements than CP cases, whose improvements ranged widely and were often on the lower end of the spectrum [9]. Our single best responder (56% improvement) would be an outlier even among CP cases, approaching results more typical of primary dystonia. Meanwhile, many of our modest responders align with most literature that shows 0–20% improvements [12].

Outcomes in dystonic cerebral palsy (CP) vary considerably and are generally less robust than those observed in primary dystonia. The literature identifies several contributing factors and predictive markers that help explain this variability.

Perhaps the strongest predictor is whether the dystonia is acquired (secondary) vs. inherited (primary). Underlying brain damage in CP (such as periventricular leukomalacia or basal ganglia lesions) likely disrupts motor pathways diffusely, limiting the therapeutic effect of GPi stimulation. Patients with normal brain MRI have been shown to have better DBS outcomes than those with significant lesions [3]. For instance, Elkaim et al. found that CP patients with normal MRI scans had higher functional outcomes than those with abnormal MRI findings [12]. In our cohort, all patients had some abnormal MRI; interestingly, Subject 9 (our best responder) had relatively milder MRI changes (no large cystic lesions, only diffuse atrophy). This anecdotal observation aligns with the notion that less structural damage = more DBS efficacy.

On the other hand, some evidence suggests that shorter dystonia duration and younger age at surgery correlate with better outcomes. A meta-regression indicated a significant inverse relationship between disease duration and percent improvement in BFMDRS—essentially, patients who lived fewer years with established dystonia improved more [3]. In our study, the two patients operated on in their early 20s (with ~15 years of dystonia) tended to improve more than those in their 30 s or 40 s who had decades of dystonia; however, our sample is too small to draw firm conclusions. We did not find a clear age-outcome correlation. Still, broadly, our results are consistent with the idea that earlier intervention might yield greater plasticity or prevent fixed musculoskeletal deformities that limit functional gains.

Regarding baseline severity, there is mixed evidence on whether initial dystonia severity predicts outcome. One recent analysis reported that higher baseline BFMDRS scores were associated with more minor percentage improvements [21], suggesting patients with milder dystonia might achieve a greater relative benefit. This might be because extremely severe dystonia (e.g., BFMDRS > 80) often coexists with irreversible orthopedic issues and contractures, blunting the functional impact of any improvement. In our series, we noted that two non-responders had among the highest baseline BFM scores (>90). While our sample is too limited for statistical correlation, this observation aligns with the idea that very severe dystonia may be less responsive (or improvements are not captured well by the scale when scores are maxed out). On the other hand, some studies have not found baseline severity to significantly predict outcome, so this factor remains to be clarified [3].

All patients in our study presented with generalized dystonia, making them typical candidates for deep brain stimulation; however, when dystonia is markedly asymmetric or accompanied by prominent dystonic tremor, adjustments in targeting may be required due to the distribution of dystonia and co-morbid movements. (e.g., GPi vs. thalamus). In our patients, choreoathetosis often accompanies dystonia. An important question is whether our patients’ functional gains were due to reduced dystonia per se versus reduction in other involuntary movements (chorea/athetosis). GPi-DBS is primarily known to suppress dystonic muscle contractions (via modulation of basal ganglia output), and its effect on choreoathetosis is less predictable [22]. The French SPIDY-2 study [23] suggested that GPi stimulation in dyskinetic CP improved dystonic posturing more than the hyperkinetic choreic movements. Similarly, a recent comparison of GPi vs. Vim (thalamic) DBS in CP found that GPi stimulation was more effective for dystonia, whereas Vim (targeting cerebellar-thalamic circuits) did not provide added benefit for choreiform movements [22]. In our cohort, we did not separately quantify chorea/athetosis, but qualitatively, caregivers reported that sustained twisting postures lessened with DBS (e.g., less rigid arching of the back), making caregiving tasks easier; however, fast, jerky movements (athetosis) persisted in many patients. Thus, we infer that the functional improvements we observed (e.g., improved feeding ability, easier transfers) were primarily due to relief of dystonic posturing and rigidity, rather than eliminating choreiform movements. In other words, DBS allowed patients to have more voluntary control and more stable core posture, which enhanced their ability to perform tasks, even though irregular movements did not disappear entirely. Future studies incorporating specific scales for chorea/athetosis could shed more light on this distinction. It may be that a combination of GPi and other targets (such as STN or Vim) is needed to address the full spectrum of dyskinesia in CP, an approach that has been explored in a few cases (dual-target DBS) with some success in controlling both dystonia and tremor [3].

All our patients received GPi-DBS, the most common dystonia target. Ongoing research is into whether the subthalamic nucleus (STN) could be an alternative target that yields equal or better results in secondary dystonia. A systematic review by Ozturk et al. (2021) compared GPi and STN-DBS across studies and found no clear overall difference in dystonia outcomes [20]. However, the experience with STN-DBS in CP is still limited, and GPi remains the gold-standard target due to its extensive track record. It is reassuring that our outcomes with GPi align with those reported elsewhere, and programming in our series was within typical parameters (130 Hz, ~3 V). There may be cases where STN-DBS is considered, especially if bradykinesia or Parkinsonian features coexist (not usually the case in CP). Our data do not directly address target selection. However, we note that two of our patients with milder dystonia and more action-induced symptoms might have hypothetically benefited from supplemental thalamic DBS for tremor; this is speculative and was not attempted in our cohort.

Finally, it is instructive to look at long-term outcomes beyond 1 year. Recent longitudinal studies indicate that improvements from DBS in dystonic CP can be sustained or even increase slightly over multiple years in some patients, while others might lose benefit over time. A 2-year follow-up study in adults with dystonic CP reported an average 46% improvement at 1 year, dropping to 39% at 2 years [6], suggesting some waning of effect or progression of disease-related issues (e.g., musculoskeletal changes) over time. Pediatric series with even longer follow-up (5–10 years) have shown that motor benefits persist, and quality of life can remain improved with significant cognitive side effects of long-term stimulation [9]. In our study, we report only 1-year outcomes; ongoing follow-up of this cohort will be valuable to see if gains are maintained at 2, 3, or 5 years. None of our patients showed tolerance or loss of benefit within the first year. Still, two periodic voltage increases were required to sustain the same effect, hinting at possible habituation that might occur in subsequent years.

## 5. Limitations

This study has several limitations. First, the sample size n = 111 is small, reflecting the rarity of adult dystonic CP cases undergoing DBS, and there was no control group. Our findings should thus be interpreted cautiously, and statistical power is limited. The heterogeneity of our sample (in terms of ages, CP etiologies, and baseline function) adds variability, but it also makes our conclusions more reflective of real-world clinical diversity. Second, as mentioned, we did not employ a dedicated clinical scale for chorea/athetosis such as the Barry-Albright Dystonia scale, which includes an athetosis component; using only the BFMDRS may not fully capture changes in all movement components. For this reason, future studies could address different methods to disentangle which aspects of movement disorder are improved by GPi-DBS. Third, our follow-up duration of 12 months is relatively short for assessing a neurostimulator intervention in a non-degenerative condition. Long-term benefits, hardware durability, and any late-emerging side effects (e.g., stimulation tolerance, infections) will require extended observation. We continue to follow these patients and plan to report their 3- to 5-year outcomes.

Additionally, while we included functional and caregiver measures, objective quality of life (QoL) measures were not directly assessed with a patient-reported outcome instrument (the FIM is clinician-rated, and CBS is caregiver-rated). In pediatric populations, instruments like CPCHILD (Caregiver Priorities and Child Health Index of Life with Disabilities) have been used to gauge caregiver-perceived QoL in CP. A recent meta-analysis confirmed that DBS in secondary dystonia leads to significant improvements in health-related quality of life (HRQoL), especially in physical health domains [10]. Future studies could incorporate such QoL scales to complement the measures we used. Nonetheless, our study’s improvements in FIM and CBS imply better day-to-day quality of life for patients and families, even if we did not quantify it with SF-36 or similar QoL surveys.

Our study did not perform formal neuropsychological assessments or mood evaluations pre-/post-DBS. Although we did not observe any overt cognitive or behavioral deterioration (and none of our patients had pre-existing cognitive impairment beyond mild intellectual disability, common in CP), subtle effects cannot be ruled out. Reassuringly, long-term pediatric DBS data have not shown significant cognitive decline attributable to stimulation [9]. Still, careful monitoring of mental–cognitive function is advisable in this population, given case reports of apathy or changes in stimulation affecting behavior.

## 6. Conclusions

In conclusion, our revised analysis demonstrates that GPi-DBS in adults with dystonic cerebral palsy can provide meaningful reductions in dystonia and associated functional improvements for some patients, while others may experience minimal benefit. The outcomes are highly individualized, reinforcing the need for careful patient selection and managing expectations. Validated functional outcome scales and caregiver burden assessments are crucial in evaluating the impact of DBS in this population, as they capture benefits beyond numerical motor scores. We found that even moderate dystonia improvements can translate into practical gains (and caregiver relief), although patients generally remain significantly disabled post-DBS. These findings lend support to the role of GPi-DBS as a component of multidisciplinary management for severe dystonic CP, particularly for those patients who have not responded to conservative treatments, but also highlight that it is not a cure-all and must be combined with ongoing rehabilitative and supportive care. Future research directions include identifying biomarkers or predictors (genetic, imaging, or physiological) that can prospectively predict which dystonic CP patients are most likely to benefit from DBS, as well as optimizing neuromodulation strategies (e.g., exploring alternative targets like STN or dual-target approaches, advanced programming paradigms, or closed-loop DBS systems) to improve outcomes. Given the lifelong nature of CP, assessments of long-term efficacy and quality of life outcomes will be essential to fully establishing the cost-benefit ratio of DBS in this condition. Our study contributes to the growing body of evidence that, with appropriate patient selection, DBS offers a safe treatment capable of modest improvements in motor function and quality of life for individuals with secondary dystonia and provides some relief to their caregivers. We advocate for a holistic outcome evaluation (motor, function, QoL, caregiver impact) in all future studies of DBS in this population, to ensure that therapeutic advances truly resonate in daily living improvements for patients and families.

## Figures and Tables

**Table 1:** Demographic and clinical characteristics of the patients.

Patient No	Sex	Age During Operation	CP Clinical Type	CP Involvement Pattern
1	F	21	chorea-atetoid, dystonic	Quadriplegia
2	F	21	chorea-atetoid, dystonic	Quadriplegia
3	F	19	chorea-atetoid, dystonic	Quadriplegia
4	M	55	dystonic	Quadriplegia
5	M	33	chorea-atetoid, dystonic	Diplegia
6	F	34	chorea-atetoid, dystonic	Diplegia
7	F	32	chorea-atetoid, dystonic	Quadriplegia
8	M	7	chorea-atetoid, dystonic	Quadriplegia
9	F	21	dystonic	Quadriplegia
10	F	6	dystonic	Quadriplegia
11	F	22	chorea-atetoid, dystonic	Quadriplegia

**Table 2:** Changes in the Burke–Fahn–Marsden Dystonia Rating Scale (BFMDRS), Functional Independence Scale (FIS), and Gross Motor Function Classification System (GMFCS) at baseline and 1 year after deep brain stimulation.

Patient No	CP-Clinical Type	BFMDRS Preoperative	BFMDRS Postoperative 1 Year	Improvement (%)	FIS Preoperative	FIS Postoperative	Improvement (%)	GMFCS Preoperative	GMFCS Postoperative
1	chorea-atetoid, dystonic	61.5	48	21.9	101	114	11.4	2	1
2	chorea-atetoid, dystonic	96	90	6.25	26	28	7.1	5	5
3	chorea-atetoid, dystonic	103	95	7.7	28	28	0	5	5
4	dystonic	68	66	2.9	91	116	24.1	2	2
5	chorea-atetoid, dystonic	47.5	39	17.8	95	116	18.1	2	2
6	chorea-atetoid, dystonic	48.5	15	69	81	99	18.1	2	1
7	chorea-atetoid, dystonic	22	8.5	60	120	126	4.7	1	1
8	chorea-atetoid, dystonic	100	98	2	47	46	−2.1	5	5
9	dystonic	55	9.5	82.7	64	126	49.2	3	1
10	dystonic	106	98	7.5	26	27	3.7	5	5
11	chorea-atetoid, dystonic	58.5	31	47	40	46	13	4	3

## Data Availability

The datasets generated during and/or analyzed during the current study are available from the corresponding author upon reasonable request.
